# Adverse Childhood Experience Chronicity and Timing: Impacts on Harsh and Nurturing Parenting in UK Black and Minority Ethnic Parents

**DOI:** 10.1007/s40653-025-00704-2

**Published:** 2025-03-29

**Authors:** Itunu Ogundiyun, Laura Katus

**Affiliations:** Institute for Lifecourse Development, School of Human Sciences, University of Greenwich, London, UK

**Keywords:** Childhood adversity, Chronic adversity, Adversity timing, Ethnic minority, Nurturing parenting, Harsh parenting

## Abstract

Adverse Childhood Experiences (ACEs) are a range of traumatic events, primarily including personal abuse, neglect, and household dysfunction, which frequently are associated with maladaptive consequences into adulthood. As such, ACEs also have the potential to adversely affect an individual’s response to their own children once they become a parent. While members of minority ethnic groups are at a higher risk of experiencing ACEs, research on how this affects parenting outcomes is limited. This study examines ACE exposure in a cohort of N = 64 Black and minority ethnic parents in the UK. We found that ACEs were positively associated with parents self-reported harsh parenting, and negatively associated with nurturing parenting. Examining ACE timing, exposure during middle childhood, but not early or late childhood or adolescence, was associated of both harsh and nurturing parenting. Examining the differential impact of ACE chronicity (i.e., prolonged exposure) and ACE frequency (i.e., number of ACEs), harsh parenting was significantly associated with ACE chronicity, whereas nurturing parenting was linked to ACE frequency. Mediation analyses showed a partial mediation of parental education for the link between ACE frequency and harsh and nurturing parenting outcomes. Our results contribute to a growing body of research highlighting the intricate interplay between early life adversity and parenting behaviours within minority ethnic communities. Findings underscore the need for targeted interventions and support systems aimed at breaking the cycle of ACEs, promoting healthier parenting practices, and ultimately fostering improved outcomes for both parents and their children in these communities. Future research should delve deeper into the specific mechanisms through which ACEs influence parenting behaviours and explore culturally sensitive approaches to mitigate their impact on minority ethnic parents.

## Introduction

Adverse Childhood Experiences (ACEs) are well documented to affect developmental (e.g., Berg et al., 2017; Cozolino, 2014; Kalmakis & Chandler, 2014; Katus et al., 2020; Pervanidou & Chrousos, 2007; Trickett et al., 2010) and, in some cases, life-long outcomes (Felitti et al., 1998). ACEs encompass a range of traumatic events, primarily including personal abuse, neglect, and household dysfunction, occurring prior to a person’s 18th birthday (Felitti et al., 1998; Widom et al., 2012), and have been linked to altered stress responses, self-regulation and neurodevelopment (Danese & McEwen, 2012; Essex et al., 2011; Shalev et al., 2013). Adverse effects can extent into adulthood (Flaherty et al., 2013; Green et al., 2010; McLaughlin et al., 2012) and can in fact show inter-generational transmission from parent to child (Lange et al., 2019). While ACEs are prevalent among the general population, marginalised and minority ethnic groups have been highlighted as particularly vulnerable (Cronholm et al., 2015; Madigan et al., 2023). However, there is a paucity of research examining how ACEs affect subsequent parenting (Bellis et al., 2019), particularly in minority ethnic groups. This study seeks to contribute to this gap, by exploring links between ACEs and parenting in a sample of Black and minority ethnic UK parents. Specifically, we examine associations between ACEs, and harsh and nurturing parenting, effects on frequency, timing and chronicity of ACE exposure, as well as the mediating role of socio-economic status.

Minority Ethnic Groups: Higher Prevalence of Childhood Adversity?

While ACEs transcend geographical, racial, ethnic, and gender boundaries, with studies documenting their occurrence in different parts of the world (Bader et al., 2007; Dube et al., 2010; Jewkes et al., 2010; Ramiro et al., 2010), individuals belonging to historically marginalised racial and ethnic backgrounds, those with low socioeconomic status, or individuals who identify as sexual minorities are more prone to experiencing ACEs (Cronholm et al., 2015; Garcia et al., 2019). Regarding minority ethnic groups, Turner & Lloyd (2003) found that African Americans reported a higher number of ACEs compared to their non-Hispanic White and Hispanic participants. A recent meta-analysis of N = 206 studies of ACE exposure highlights that belonging to a minority ethnic group constitutes an important predictor of having experienced 4 + ACEs (Madigan et al., 2023). Further, a majority of studies included in the meta-analysis (83.5%) were conducted in North America (Madigan et al., 2023), highlighting the need to further investigate the prevalence and consequences of ACEs in minority ethnic groups outside the US.

### ACE Exposure and its Effects on Parenting

Outcomes associated with ACE exposure appear to exhibit relative consistency across diverse backgrounds (Hughes et al., 2017; Massetti et al., 2020). Individuals exposed to ACEs may exhibit behavioural disorders, anxiety, and lower self-rated health in adulthood (Chan & Yeung, 2009; Chartier et al., 2010). Studies have also shown the overall impact of ACEs pertaining to parenting stress and behaviours (Chung et al., 2009; Steele et al., 2016; Anda et al., 2006; Bert et al., 2009; Bryan, 2019; Hughes & Cossar, 2016). In a study of mothers with a history of childhood abuse, McDonnell and Valentino (2016) reported associations between maternal childhood maltreatment, household dysfunction, and elevated levels of maladaptive socioemotional symptoms in their infants. Examining specific parenting behaviours, Bert et al. (2009) observed a significant association between maternal self-report of emotional abuse during childhood and reduced levels of responsivity and empathy towards their six-month-old infants. A wealth of research indicates that warm and supportive parenting plays a critical role in fostering resilience and reducing the likelihood of negative life outcomes (Howell et al., 2021; Moe et al., 2018; Repetti et al., 2002). Adverse family dynamics, such as frequent conflict, distant parent–child relationships, and inconsistent or harsh disciplinary practices on the other hand have been associated with adverse effects on children's cognitive and emotional development (Bevilacqua et al., 2021; Lange et al., 2019). Equally, the use of positive parenting practices becomes particularly significant for children growing up in families facing challenging circumstances or stressors, such as economic difficulties, parental divorce, or parental illness (Lomanowska et al., 2017), since positive parenting may buffer against and thus alleviate potential adverse effects of ACEs on parenting behaviour.

ACEs have been linked to higher levels of parenting stress, which can impact on parental attitudes and behaviour toward their children (Lange et al., 2019; Steele et al., 2016). Parents who experienced more than two ACEs are more likely to struggle with bonding with their children, which can lead to neglectful behaviour (Murphy et al., 2014). Similarly, mothers who experienced abuse as children are reported to be less sensitive to their children, with parenting stress found to influence the level of responsiveness (Pereira et al., 2012). In part these findings might be accounted for by the potential for intergenerational transmission of parenting, which occurs when parents who experienced abuse during their own childhood are currently raising a maltreated child in their family (Belsky et al., 2005; Conger et al., 2003). Hereby, the continuing pattern is not specific to the perpetrator; rather it describes the transfer of overall risk for maltreatment from parent to the child, regardless of the probability that either or both parents themselves engage in the abuse (Scaramella et al., 2008). A potential mechanism by which intergenerational transmission might play out is via parental mental health: a wealth of research from large-scale, representative cohorts has highlighted how adversity in childhood (Newbury et al., 2018) and adolescence (Latham et al., 2022) is associated with higher likelihood of psychopathology in adulthood. Indeed, some evidence suggests that links between parental ACEs and children’s adverse health and behavioural problems are amplified, where parents themselves experience mental health problems, further highlighting the importance of this mechanistic link (Schickedanz et al., 2018).

### ACE Timing, Frequency, and Chronicity

While existing studies have primarily examined specific types of childhood trauma and associations with later parenting practices, it is important to consider the timing at which these traumatic events occur, as they can vary significantly. In part this diversity in associated outcomes of early exposure to childhood trauma and adversity may be attributed to the nature of the specific adverse experience: hereby, it needs to be noted that childhood trauma refers to a narrower range of experiences, namely those that pose active threat to a child and therefore are highly likely to elicit a stress response (Browne & Winkelman, 2007). The concept of early adversity however is a broader one, encompassing any deviation of an expectable environment, and thus also encompassing exposures that may not activate a physiological stress response but still are associated with subsequent adverse consequences (Nelson & Gabard-Durnam, 2020). Extensive research highlights the widespread occurrence of ACEs and their interconnected nature, emphasising their cumulative effects (Anda et al., 2010; Dong et al., 2004; Felitti et al., 1998). However, exposure to a single ACE does not always indicate adverse outcomes, and children who experienced only a single risk factor tend to exhibit minimal or no long-term maladaptive effects (Anda et al., 2006; Bryan, 2019). Nonetheless, exposure to a single ACE is uncommon, and children who experience multiple ACEs are categorised to be at higher risk, and vulnerable to serious and long-lasting adverse effects (Bryan, 2019; Kalmakis & Chandler, 2014; Rutter, 1979). Furthermore, there is strong evidence indicating that timing of adversity matters in predicting adverse outcomes (Schroeder et al., 2020).

ACEs frequently occur chronically and recurrently (Schroeder et al., 2020). Theoretically, Gabard-Durnam & McLaughlin (2019) highlight how repeated ACE exposure might take effect on development via a range of mechanisms, including 1) accumulation of risk factors (i.e., each adverse experience, as well as different kinds of risk factors increases the propensity of adverse outcomes) and 2) dose–response effects (i.e., more severe adversities leading to more severe or prolonged disruptions to development, causing greater impact. Because of the increased risk of psychopathology and physical illness linked with chronic adversity, it is vital to distinguish between chronic and acute experiences in childhood (Jirapramukpitak et al., 2011; Schafer & Ferraro, 2011). While ACEs can manifest as a single traumatic event (acute), chronic exposure to hardship is more characteristic for ACE exposure (van der Kolk, 2005). According to Masten and Cicchetti (2010), ACE chronicity can lead to the gradual accumulation of negative coping mechanisms, whereas single instances of adversity may require short-term strategies and do not have lasting effects on development. Thus, most studies conclude that chronicity is a common feature of ACEs associated with more negative and longer-lasting consequences (Chartier et al., 2010; Dunn et al., 2017; Dierkhising et al., 2019; Schroeder et al., 2020). Work by Naar-King and colleagues, (Naar-King et al., 2002) further investigated the influence of abuse duration, finding longer durations of physical abuse to be connected with increased feelings of anxiety and depression, as well as increased likelihood for prolonged periods of physical harm. In addition, Manly et al. (2001) concluded that children who were subjected to chronic maltreatment during early childhood exhibited the poorest behavioural outcomes compared to cases involving maltreatment confined to a single developmental period. Recent studies investigating poly-victimised adolescents exhibited the most severe symptoms for internalised and externalised difficulties as well as post-traumatic stress disorder (Dierkhising et al., 2019). Additionally, Hawes et al. (2021) reported that the chronicity of ACEs was related to current symptoms of child psychopathology. This agrees with an earlier longitudinal study by Thompson et al. (2015), which found that individuals with chronic ACEs exhibited increased health concerns compared to those with low ACE exposure. However, in a study utilising data from the Fragile Families and Child Wellbeing Study, Schroeder et al. (2020) found that chronic adversity, characterised by two or more ACEs at ages one, three, and five, did not show a significant association with behaviour problems in middle childhood. Next to chronicity, conflicting findings may be accounted for the timing of ACE exposure.

Previous research on ACEs has primarily used an overall risk approach, with evaluation focusing on the entirety of adversities suffered during childhood. This approach, however, contradicts an expanding body of literature that highlights the intricate transactional mechanisms through which ACEs impact mental health during childhood and adolescence (McLaughlin & Sheridan, 2016). Regarding mechanistic accounts for links between adversity exposure and outcomes, Gabard-Durnam and McLaughlin (2019) highlight that timing of exposure matters: where there is a co-occurrence of adversity with critical and sensitive developmental periods this may mean that developmental trajectories are altered in the long term. Notably, scholars have investigated unique pathways of children's outcomes according to the developmental timing of ACE exposure (Schroeder et al., 2020; Thornberry et al., 2001). Investigations into the timing of ACE exposure indicate that early childhood, encompassing infancy through preschool age, represents a particularly vulnerable period (Kotch et al., 2008). Appleyard et al. (2011) discovered that children exposed to a greater number of ACEs during early childhood (zero—five years) exhibited more externalising problems at age 16 compared to those with fewer ACEs during the same period. More recently, a longitudinal study revealed that ACEs experienced during early childhood (ages zero to three) were associated with behaviour problems in middle childhood, even after considering adversity at other ages and cumulative adversity (Schroeder et al., 2020). Another study found that individuals who had experienced maltreatment in early childhood exhibited twice as many symptoms of depression and post-traumatic stress disorder (PTSD) compared to those who experienced maltreatment in later developmental periods (Dunn et al., 2017). Alternative findings have suggested the unique effects of ACEs in middle childhood. Supporting this notion, Keiley et al. (2001) found that an earlier onset of maltreatment significantly correlated with increased problem behaviour during middle childhood. Also, research by Riem and colleagues (Riem et al., 2015) observed a relationship between hippocampal volumes and the timing of childhood maltreatment, with greater volume reductions for those reporting maltreatment at older ages. Similarly, another study found that adversity experienced in middle childhood (ages five to 11), but not early adversity (ages zero to five), was associated with elevated cortisol levels during adolescence (Bosch et al., 2012). In a deeper investigation examining the connection between neglect or abuse and aggression, Kotch et al. (2008) focused on early childhood (ages zero to two) and middle childhood (ages three—eight years). Their findings indicated that neglect in early years was linked to aggression scores, whereas abuse in early years, or later years as well as later neglect, did not. This implies that effects vary not just by type of abuse but also by the developmental timing of exposure. The mixed findings underscore the necessity for further research on the timing of adversity exposure and its significant implications for theoretical models exploring the contributions of the environment as well as early life experiences to future life outcomes. It also underscores the importance of capturing evidence not only on the type but also on the particular period of adverse experiences in assessment methods designed to identify crucial factors affecting adult behaviours. They also provide evidence suggesting that the influence of ACEs on a growing child may vary depending on the ages at which adverse environments are encountered.

### The Present Study

This study aims to assess the influence of ACE frequency (number of ACEs across development), ACE chronicity (number of developmental periods for which ACEs were reported) and timing (number of ACEs for early, middle and late childhood, and adolescence) on parenting practices. The study utilises classifications based on the US National Research Council Panel and the Adverse Life Experiences Scales (ALES) to operationalize the developmental timing of ACE exposure (early childhood—birth to age 5, middle childhood—ages 6–8, late childhood—ages 9–12, adolescence – ages 13–18). Our first aim is to assess whether the extent of ACE exposure is associated with parenting in a sample from a previously understudied population of Black and ethnic minority parents in the UK. We will then examine the matter of ACE timing, by testing whether ACEs experienced during early and middle childhood differentially relate to parenting outcomes. Next, we will examine the differential impact of ACE timing and chronicity for parenting outcomes. Lastly, we will examine potential socio-economic mediators (parental education and household income).

### Hypotheses

- 1.ACE exposure will be positively associated with harsh parenting.
- 2.ACEs experienced during early and middle childhood will have the exhibit strongest associations with later parenting.
- 3.Both ACE frequency and chronicity will show independent associations with parenting outcomes.
- 4.Parental education and income levels will mediate the relationship between ACEs and harsh parenting practices.

## Methods

### Participants

Participants were recruited via online adverts on social medial and via the Qualtrics Survey Management System. To be included in analyses participants needed to 1) be over the age of 18, 2) have at least one child between the ages of 5 and 15 years, 3) report their ethnicity as belonging to a minority ethnic group. Furthermore, participants had to contribute at least 50% of items on both main outcome measures of the study (see ‘Measures’ below). The study protocol was approved by the School of Human Sciences Research Ethics Panel ad the University of Greenwich.

### Procedures

Data were collected via Qualtrics between March and July 2023. Participants who provided informed consent were screened with regard to the eligibility criteria and completed the self-paced online questionnaire. Following completion, participants were provided with information of their right to withdraw and contact details of the lead researchers. Due to the sensitive nature of the questions, participants were also provided with contact details to a mental health helpline should they experience significant distress after completing the survey.

### Measures

*Demographic data.* Participants provided data on various demographic variables, including age, gender, marital status, ethnicity, highest education level and pooled household income.

*Adverse Childhood experiences (ACEs).* To assess ACEs, we drew on the Adverse Life Experience Scale (ALES, Hawes et al., 2021), a novel self-report instrument designed for parents and caregivers. The ALES incorporates the ACE inventory developed by (Felitti et al., 1998), along with modified items encompassing aspects such as exploitation or victimisation by peers, peer isolation, discriminatory experiences, and exposure to violence or war. The caregiver questionnaire comprises 24 dichotomous (yes/no) items that capture various ACEs, including, ‘*Have you felt lonely, or been rejected or excluded by peers*?’, ‘*Have you been separated from or lost someone who you depended on for love or security? (e.g., due to foster care, abandonment, immigration, war, severe illness, or death*)’ and *‘Have you seen another person seriously injured or killed, or have repeatedly heard about others getting hurt or killed?’*. The parent scale of the ALES has been found to exhibit good to high internal consistency (α = 0.86), and test–retest reliability over a 3–6 week period (ICC = 0.88) in its original validation sample of Australian families (Hawes et al., 2021).

*Parenting practices.* To assess harsh as well as nurturing parenting practices, we drew on the widely used Alabama Parenting Questionnaire (APQ, Frick et al., 1999), a self-report questionnaire consisting of 42 items designed to assess current parenting practices. It comprises five sub-scales: Positive Parenting – 6 items (e.g., *“You reward or give something to your child for obeying you or behaving well?”*); Poor Monitoring – 10 items (e.g., *“You get so busy you forget where your child is and what he/she is doing?”*); Inconsistent Discipline – 6 items (e.g., “You threaten to punish your child and then do not actually punish him/her?”); Involvement – 10 items (e.g., “You play games or do other fun things with your child?”) and Corporal Punishment – 3 items (e.g., “You spank your child with your hand when he/she has done something wrong?”). The questionnaire included an additional seven items to evaluate aspects of discipline other than corporal punishment. The inclusion of these items was intended to avoid a negative bias towards corporal punishment items (Święcicka et al., 2019). Responses are recorded on a Likert-type scale ranging from Never (1) to Always (5). From the questionnaire’s subscales indices of harsh parenting (corporal punishment, inconsistent discipline and discipline items) and nurturing parenting (positive parenting and involvement subscales) were obtained. Previous studies have reported acceptable internal consistency reliabilities for the five scales ranging from α = 0.63 to 0.80 (Frick et al., 1999; Shelton et al., 1996). The APQ demonstrates robust psychometric properties and has been shown to possess criterion validity in distinguishing between clinical and non-clinical populations (Dadds et al., 2003).

## Statistical Analyses

The following indices were obtained and used in all further analyses:

1. ACE frequency: the total number of reported ACEs across all 23 items of the ALES (higher = greater ACE exposure).
2. ACE chronicity: the number of age points during which any ACE were reported (higher = more chronic exposure).
3. Early childhood ACEs: sum of ACEs reported between 0–5 years of age.
4. Middle childhood ACEs: sum or ACEs reported between 6–8 years of age.
5. Late childhood ACEs: sum of ACEs reported between 9–12 years of age.
6. Adolescent ACEs: sum of ACEs reported between 13–18 years of age.

Based on these outcome measures, we first examined associations between total ACEs and self-reported scores on the harsh and the nurturing parenting subscales via linear regression models. Where an overall effect for ACE sum scores and parenting was found, we followed up these analyses by examining whether ACE exposure during early, middle or late childhood was found to be most strongly associated of parenting via linear regression models. Next, we examined the relative impacts of ACE chronicity vs frequency on parenting. To this end we entered ACE frequency and ACE chronicity into linear regression models. To account for potentially protective effects, we concluded our analyses by conducting cross-sectional mediation analyses, to examine the potential mediating effects of education level and income on the link between childhood adversity and parenting practices.

## Results

Descriptive statistics can be found in Table 1. The majority of participants were between the ages of 25–44 (79.7%), majority female (59.4%) and married/partnered (89.1%). Further, all participants were of either Black British (79.9%) or other/mixed ethnicity (20.1%). The majority of the sample had obtained university degrees (96.9%) and reported a wide range in household income levels, ranging from less than £10,000 to over £100,000 per year. In terms of parents’ ACE exposure, 93.2% of parents had reported at least one ACE, and 65.8% reported four or more ACEs. ACE frequency scores ranged from 0–15, with a mean of 5.39. Responses on the APQ also showed a considerable range for Harsh Parenting (3–21, mean = 11.2) and Nurturing Parenting (30–63, mean = 48.44).

**Table 1:** Descriptive statistics

Variable	Frequency	
	*n*	%
Gender		
Female	38	59.4
Male	26	40.6
Age		
18 – 24	2	3.1
25 – 34	15	23.4
35 – 44	36	56.3
45 – 54	9	14.1
55 – 64	2	3.1
Marital status		
Single	4	6.3
Married/partnered	57	89.1
Divorced	2	3.1
Separated	1	1.6
Ethnicity		
Black British	51	79.9
Mixed Ethnicity/Other	13	20.1
Highest educational level		
Associate degree/Some college	2	3.1
Bachelor’s degree	17	26.6
Master’s degree	24	37.5
Doctoral degree	3	4.7
Professional degree (MD, JD)	15	23.4
Annual Household Income		
Less than £10,000	15	23.4
£10,000—£19,000	7	10.9
£20,000—£39,000	7	10.9
£40,000—£59,000	5	7.8
£60,000—£79,000	8	12.5
£80,000—£99,000	8	12.5
£100,000 – above	14	21.9
	X̄ ± SD	range
Adverse Life Experiences Scale (ALES)		
ACE frequency	5.39 ± 3.632	0–15
ACE chronicity	1.88 ± .823	0–3
ACE early childhood	1.933 ± 1.751	0–6
ACE middle childhood	2.29 ± 1.677	0–7
ACE late childhood	3.4 ± 2.499	0–10
ACE adolescence	4.67 ± 3.146	0–12
Alabama Parenting Questionnaire (APQ)		
Harsh Parenting	11.2 ± 4.202	3–21
Nurturing Parenting	48.44 ± 7.346	30–63

Both the ALES and the APQ showed acceptable to good internal consistency (ICC_ALES_ = 0.76, APQ, ICC_APQ_ = 0.71).

### ACE Exposure and Parenting Practices

**Fig. 1:**
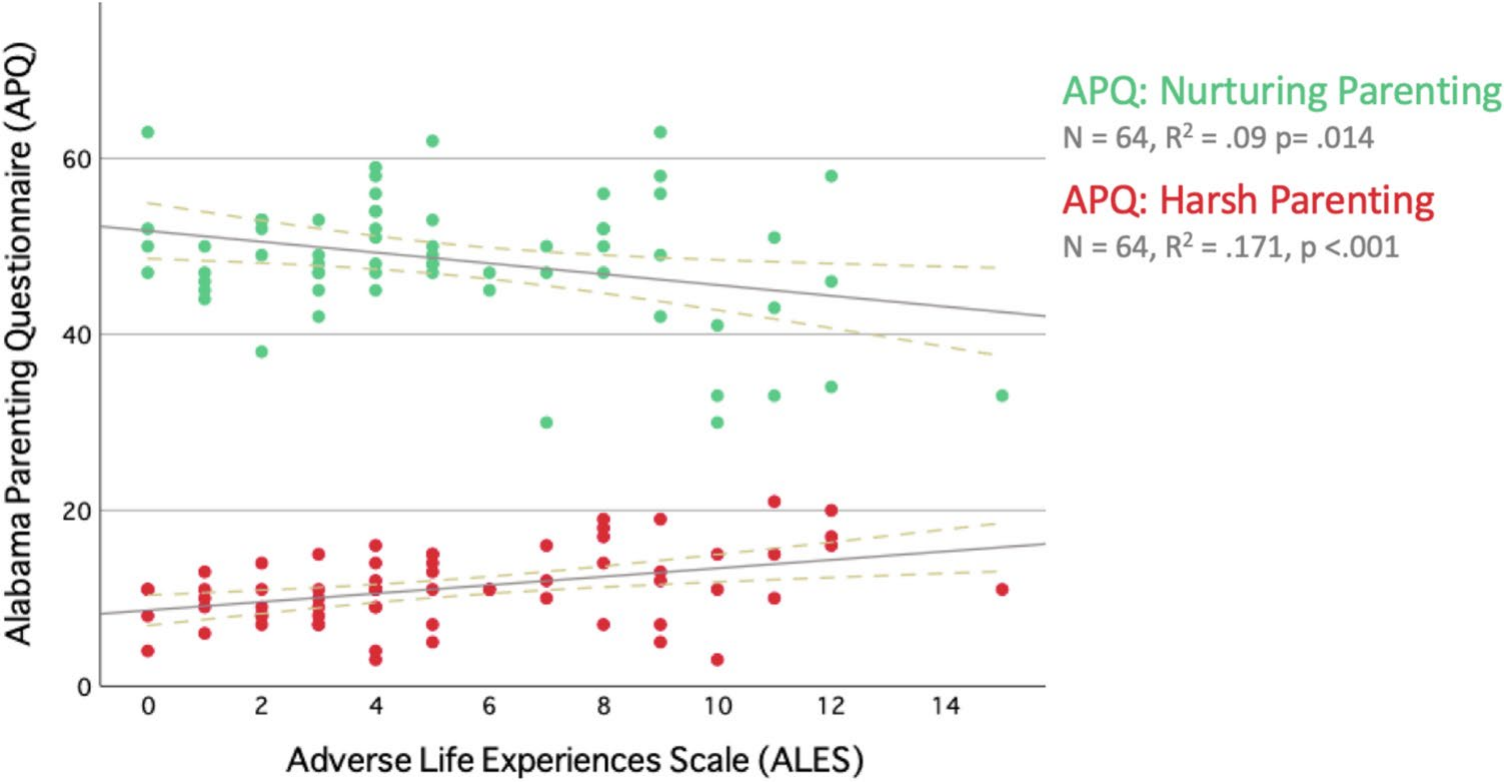
Correlations between Alabama Parenting Questionnaire and Adverse Life Experiences Scale (ALES). There was a significant negative correlation between childhood adversity and subsequent nurturing parenting (green), indicating that higher ACE exposure was linked to lower nurturing parenting scores. Childhood adversity was positively correlated with subsequent harsh parenting (red), indicating greater harsh parenting scores for those participants with greater ACE exposure

To examine whether ACEs across childhood and adolescence (ages 0–17 years) were associated with positive and harsh parenting, we ran separate linear regression models with outcomes harsh and nurturing parenting. ACE scores were significantly associated with harsh parenting (β = 0.5, SE = 0.132, p < 0.001, R^2^ = 0.189), with higher ACE scores being associated with higher harsh parenting scores, and nurturing parenting (β = −0.503, SE = 0.235, p = 0.037, R^2^ = 0.069), with higher ACEs being associated with lower nurturing parenting scores. While the predicted positive association between ACEs and harsh parenting was found, we also found ACEs to be negatively associated with nurturing parenting. Correlations based on these regressions are visualised in Fig. 1.

### ACE Exposure During Early, Middle and Late Childhood and Adolescence

We assessed whether ACEs were most strongly associated depending on whether they had been encountered in early, middle or late childhood by entering the sum of ACEs for early (0–5 years) middle (6–8 years) and late (9–12 years) childhood and adolescence into regression models separately for harsh and nurturing parenting. We found an effect of middle childhood ACE exposure on harsh parenting (β = 1.596, SE = 0.473, p = 0.005) and nurturing parenting (β = −2.938, SE = 1.289, p = 0.040). None of the other age bands was significantly associated with parenting behaviours (p all > 0.290).

### ACE Frequency and Chronicity and Parenting Outcomes

**Fig. 2:**
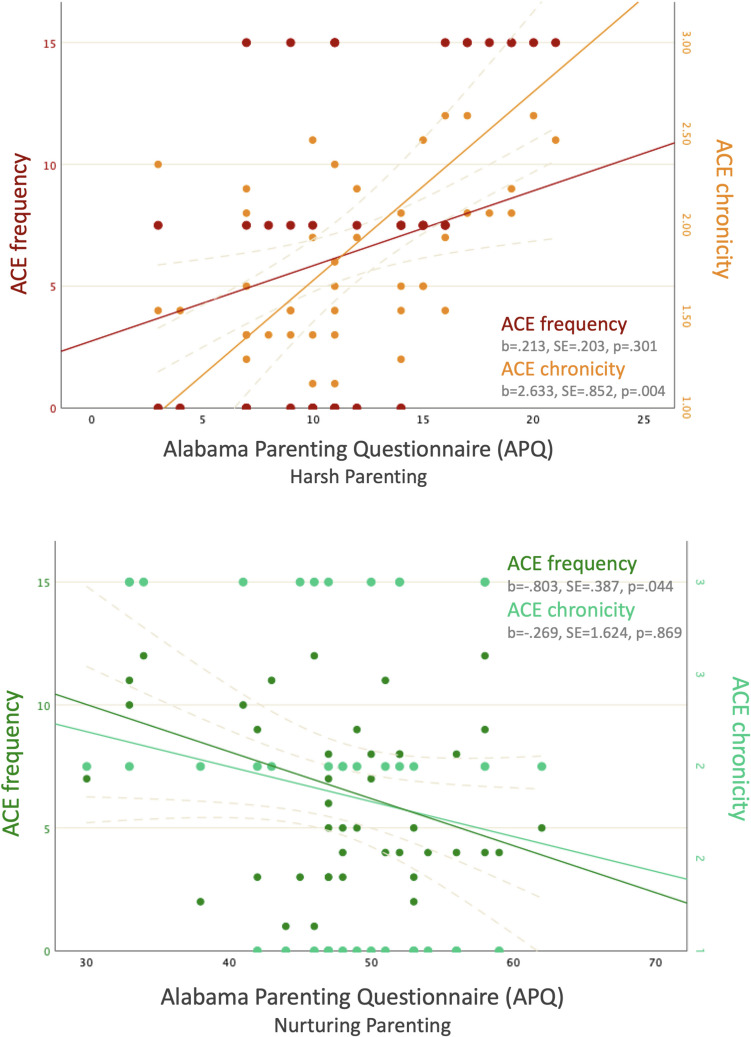
Associations of ACE frequency and ACE chronicity with APQ Harsh Parenting (top) and Nurturing Parenting (bottom) subscales. Harsh Parenting is significantly associated with ACE chronicity, whereas Nurturing Parenting is significantly associated with ACE frequency

Assessing relative associations of ACE frequency vs chronicity and later parenting, we entered overall ACE scores (summed across all developmental periods) and ACE chronicity scores (number of developmental periods during which ACE was encountered) into regression models, with harsh parenting and nurturing parenting as separate outcomes. For harsh parenting, we found a significant association with ACE chronicity (β = 2.633, SE = 0.852, p = 0.004), whereas ACE frequency, when accounting for ACE chronicity, was not significantly associated with harsh parenting (β = 0.213, SE = 0.203, p = 0.301). For nurturing parenting, we found the opposite pattern, whereby ACE frequency was associated significantly with nurturing parenting (β = −0.803, SE = 0.387, p = 0.044), whereas chronicity was not (β = −0.269, SE = 1.624, p = 0.869). These results indicate that both ACE frequency and chronicity matter for parenting outcomes, with ACE frequency impacting more on nurturing care, whereas ACE chronicity impacts more on harsh parenting. Results are visualised in Fig. 2.

### Mediating Roles of Parental Education and Household Income

**Fig. 3:**
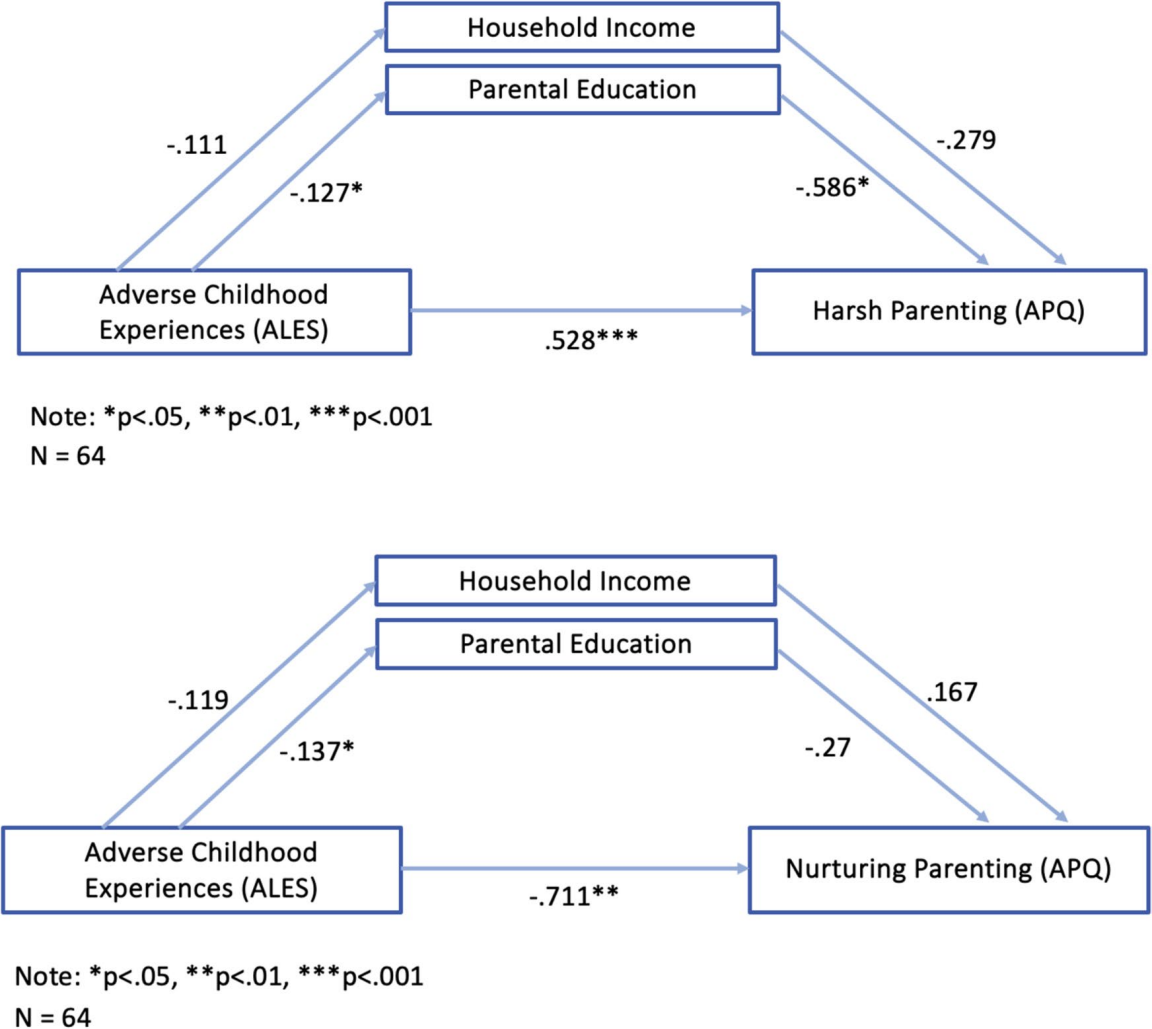
Associations between ACEs and Harsh Parenting (top) and ACEs and Nurturing Parenting (bottom). A direct association was found between ACEs and Harsh Parenting, which was partially mediated by Parental Education. A direct link was also found between ACES and Nurturing Parenting, which again was partially mediated by parental education

Lastly, we ran cross-sectional mediation analyses examining whether the link of ACE frequency and parenting practices was mediated by parental education and household income (Fig. 3). Mirroring regression analyses presented above, there were direct effects between ACE frequency and Harsh Parenting (β = 0.528, SE = 0.137, p < 0.001), as well as ACE frequency and Nurturing Parenting (β = −0.711, SE = 0.258, p = 0.008).

The link between ACE frequency and Harsh Parenting was partially mediated by parental education, as was the link between ACE frequency and Nurturing Parenting.

## Discussion

ACEs constitute a prevalent and interconnected set of factors with far-reaching implications (Choi et al., 2020). A substantial body of research highlights that the consequences of childhood adversity extend beyond the formative years (Shrira, 2012; Turner & Butler, 2003). Thus far, limited attention has been directed toward understanding the mechanisms and determinants underpinning the formation of parenting (Kilpatrick et al., 2013; Kotchick & Forehand, 2002), especially in minority ethnic parents. Moreover, research investigating the timing and chronicity of a parent’s early exposure to ACEs in their present parenting practices remains limited (Chung et al., 2009; Steele et al., 2016). This study advances the emerging line of interest within the ACEs literature that investigates the complex interplay between ACEs and parental behaviour.

The first objective of this research was to investigate the potential impact of childhood adversity levels on individuals' parenting practices in later stages of life. The majority of parents (93.2%) reported at least one ACE, with more than half (65.8%) falling above the commonly used criterion of four or more ACEs. This observation aligns with a recurrent theme in prior studies, which consistently demonstrate that a significant proportion of adults with ACE histories have encountered multiple ACEs (Chapman et al., 2013; Ho et al., 2020; Dong et al. 2004). In line with our hypothesis, we found that more frequent exposure to ACEs to be associated with higher harsh parenting behaviours. While we did not have a specific hypothesis regarding nurturing parenting, we found the opposite association, whereby more frequent ACEs were linked to lower nurturing parenting outcomes. These results link in with prior research indicating a strong association between childhood maltreatment and reduced parental self-efficacy (Caldwell et al., 2011).

In terms of the timing of ACE exposure, middle childhood, but not other age bands, was linked with higher harsh and lower nurturing parenting. While we hypothesised that exposure during early and middle childhood would be most strongly linked with parenting outcomes, this hypothesis was thus only partially supported by the data. This further adds to the mixed literature on the timing of ACEs and their subsequent outcomes. While several studies highlight middle childhood as a period of increased vulnerability to the effects of adversity (Keiley et al., 2001; Riem et al., 2015; Bosch et al., 2012; Kotch et al., 2008), effects are likely not confined to any one period, and early childhood and exposure during early (Dunn et al., 2017), as well as late childhood and adolescence has also been flagged as a risk factor. Theoretical accounts on early critical and sensitive periods might suggest increased vulnerability during the early years (Gabard-Durnam & McLaughlin, 2019). However, as the majority of ACE studies employ retrospective-recall designs, reports from early childhood might be less reliable as a result. Some studies highlight the relevance of early childhood, at least for short-term longitudinal outcomes (e.g., Schroeder et al., 2020). While challenging to employ, prospective longitudinal designs, or designs considering multiple informants (e.g., incorporating records from foster parents or police records, where applicable) are needed to add to this research.

Examining the relative impact of ACE frequency and chronicity, we found opposite patterns for harsh and nurturing parenting: harsh parenting was associated with greater ACE chronicity, whereas nurturing parenting was linked with greater ACE frequency. One explanation for this finding may be that overall ACE exposure might mean parents are less able to engage positively with their child, while harsh and punitive parenting may be the result of chronic exposure to adversity throughout development. While knowledge on differential effects of cumulative adversity vs chronicity on parenting is limited, some authors highlight differences with regard to developmental outcomes: for example, Schroeder et al. (2018) found that timing and duration of ACEs predicted internalising and externalising problems in middle childhood, over and above effects accounted for by ACE frequency.

The relatively high prevalence of reported ACEs within this sample of minority ethnic participants is in line with existing research (Merrick et al., 2018; Strompolis et al., 2019; Taillieu et al., 2014; Slopen et al., 2016). This suggests that risk of exposure to early adversity may indeed vary along racial and ethnic lines (Alegría et al., 2013). It is essential, however, to approach this observation with discretion as a higher prevalence of ACEs among individuals of Black ethnicity does not automatically translate into adverse or negative behavioural outcomes or parenting behaviour. While ACEs may indeed be more common among minority participants, it is noteworthy that when considering economic status, impoverished White families report a higher aggregate exposure to ACEs than their Black and Hispanic counterparts (Mersky & Janczewski, 2018; Slopen et al., 2016). This implies the influence of confounding factors beyond ethnicity alone, suggesting that race does not singularly determine the risk or likelihood of ACE exposure. Indeed, our mediation models underline this, as parental education partially mediated the association of ACE frequency and parenting behaviours for both harsh and positive parenting. These findings underscore the importance of conducting more comprehensive research that considers the intersection of ethnicity, socioeconomic status, and other contextual factors when assessing the impact of ACEs. Furthermore, the influence of cultural factors emerged as a significant consideration. Cultural norms and perceptions of parenting behaviours can vary widely, impacting the interpretation of what constitutes warm or harsh parenting. It is essential to acknowledge these cultural differences and their implications for understanding the effects of ACEs on parenting practices within diverse populations.

Furthermore, it is worth noting that previous studies examining ACE exposure and its impact on parenting stress may not have fully accounted for the roles of protective factors, such as social support and the educational level of parents (Danese, 2020; Hays-Grudo & Morris, 2020). Participants in this current study possessed a high level of education, with almost all (96.9%) reporting having obtained a university or postgraduate degree. Indeed, despite the overall high levels of education and the resulting limited variance within the sample, we found level of education to partially mediate links between ACEs, and harsh and nurturing parenting. This is supported by existing research indicating that maternal education can play a key role in mitigating the risks associated with adverse experiences (Rieder et al., 2019; Walker et al., 2011).

## Limitations & Strengths

There are several noteworthy limitations inherent in the present study. Primarily, this study heavily relied on a single source of reporting for all measurements. Specifically, parents were tasked with self-assessing their parenting behaviours and retrospectively rating their childhood exposure to ACEs. Consequently, it is important to note that the findings of this study may have been influenced by shared source variance. Additionally, it is well-established that parents' self-ratings of their parenting behaviours often do not correlate directly with observed parenting behaviour (Bailey et al., 2012), and future research should consider obtaining more objective, naturalistic observations of parent–child interactions. Additionally, the study was constrained by a relatively small sample size that did not provide adequate statistical power to investigate additional factors of interest (e.g., parental age, child age, parental mental health). Recent studies have begun to highlight potential mechanisms through which ACEs may exert their influence on parenting attitudes and behaviours, which should be considered for future research and include parental mental health or stress-induced interactions between a parent and their child which can serve as triggers for unresolved childhood trauma (Hays-Grudo & Morris, 2020; Lieberman et al., 2005). Furthermore, the cross-sectional design represents a limitation, and future prospective longitudinal research could help corroborate whether results of our analyses, especially our mediation analyses, hold in a longitudinal design. While ACEs are frequently assessed retrospectively, our results need to be viewed in light of possible recall bias. This is particularly true given the chronicity measures, in which participants not only need to recall whether an ACE was encountered, but also need to recall the approximate timing for different experiences. Not only may this introduce additional bias, but may in fact may particularly bias recall for some (e.g., infancy and early childhood) more so than that for later developmental periods. However, research indicates that retrospective responses regarding ACEs tend to exhibit stability over time (Dong et al., 2004; Hardt & Rutter, 2004). Additionally, it is worth noting that some instances of maltreatment may not have been self-identified, introducing another potential source of bias in our study concerning retrospective self-reports of childhood maltreatment. However, there is an accumulating body of evidence suggesting that concerns about the reliability of retrospective reports of trauma may be overstated.

Despite these limitations, the present study has some relative strengths that significantly contribute to the literature on ACEs and parenting. Firstly, our sample contributes to the scarce data on minority ethnic parents, who remain underrepresented despite the fact that heightened vulnerability has been proposed. This perspective has the potential to enhance the development of screening and intervention strategies geared towards reducing early adversity exposure (primary prevention), mitigating resultant pathology (secondary prevention), and aiding individuals already experiencing adverse effects (tertiary prevention and treatment) within a more culturally diverse context. Our findings underscore the importance of adopting a comprehensive framework when interpreting the connection between ACEs and parenting practices. Future research endeavours would benefit from incorporating a broader array of variables, employing larger and more diverse participant samples, and adopting longitudinal study designs to capture the evolving nature of parenting behaviours over time. Such approaches would enhance our understanding of the intricate interplay between adverse childhood experiences and parenting practices in a more nuanced and contextually rich manner.

## Conclusion

Understanding the relationship between frequency, chronicity and timing of childhood adversity and subsequent parenting, holds the potential to provide insights into the mechanisms through which trauma, adversity, and stress during specific developmental periods influence parenting practices. Such insights could pave the way for targeted interventions, tailored to the moderating influence of ACE exposure. The study of ACEs and their influence on parenting is a complex and multifaceted endeavour. It transcends the boundaries of childhood and adulthood, encompassing factors such as timing, chronicity, cultural context, and individual resilience. Here, we highlight the importance of considering timing and chronicity of adversity in addition to more traditionally employed measures of ACE frequency. Future research endeavours must consider the multifaceted nature of ACEs and parenting practices. This entails incorporating diverse samples, employing longitudinal designs, and accounting for cultural variations. Such efforts will not only enhance our comprehension of the relationship between different aspects of adversity exposure, but also inform the development of more effective interventions and support systems for individuals and families impacted by ACEs. This research suggests that when examining ACEs in research and practice, it is also important to consider protective and resilience-promoting factors. Doing so can help researchers to better understand developmental trajectories and outcomes and can aid practitioners gaining a more comprehensive understanding of early experiences. It reaffirms the importance of adopting a nuanced and culturally sensitive approach when studying the profound influence of childhood adversity on parenting practices which will aid the development of those that are more effective for different populations.

## Data Availability

All data underlying this submission are provided in anonymised format on the Open Science Framework: https://osf.io/gqe8h/
